# Supplementary material for: Influenza A Virus Infection Reactivates Human Endogenous Retroviruses Associated with Modulation of Antiviral Immunity
Source: Viruses. 2022 Jul 21;14(7):1591. doi: 10.3390/v14071591 (PMC9320126; doi:10.3390/v14071591)
Supplement: Supplementary file 1 [file viruses-14-01591-s001.zip › MotifAnalysis.zip!/MotifOutput_SC35M/homerResults/motif1.similar.html]

motif1

## Information for motif1

C
G
T
A
A
C
G
T
C
T
G
A
A
G
T
C
C
G
T
A
A
G
T
C
A
G
T
C
G
T
C
A
T
A
C
G
A
C
G
T
  
Reverse Opposite:  

C
G
T
A
A
T
G
C
A
C
G
T
A
C
T
G
A
C
T
G
A
C
G
T
A
C
T
G
A
G
C
T
C
G
T
A
A
C
G
T
  

|  |  |
| --- | --- |
| p-value: | 1e-20 |
| log p-value: | -4.701e+01 |
| Information Content per bp: | 1.918 |
| Number of Target Sequences with motif | 13.0 |
| Percentage of Target Sequences with motif | 31.71% |
| Number of Background Sequences with motif | 208.5 |
| Percentage of Background Sequences with motif | 0.44% |
| Average Position of motif in Targets | 130.1 +/- 57.4bp |
| Average Position of motif in Background | 105.2 +/- 65.0bp |
| Strand Bias (log2 ratio + to - strand density) | 10.0 |
| Multiplicity (# of sites on avg that occur together) | 1.17 |
| Motif File: | file (matrix) reverse opposite |

### Similar de novo motifs found

|  |  |  |  |  |  |  |  |
| --- | --- | --- | --- | --- | --- | --- | --- |
| Rank | Match Score | Redundant Motif | P-value | log P-value | % of Targets | % of Background | Motif file |
| 1 | 0.679 | C G T A C G T A A G T C C T G A A G T C C G T A A G T C A G T C C G T A C G T A A C G T A G T C | 1e-15 | -35.560128 | 24.39% | 0.36% | motif file (matrix) |
| 2 | 0.605 | G T C A C G T A A C G T C T A G A T C G C G T A A G T C A G T C | 1e-14 | -32.660149 | 41.46% | 3.37% | motif file (matrix) |
| 3 | 0.685 | A G T C C G T A A G T C A G T C A G T C A C T G A C G T C G A T | 1e-13 | -32.098411 | 41.46% | 3.49% | motif file (matrix) |
| 4 | 0.646 | A G T C C G T A A G T C A G T C C G T A C G T A A C G T A G T C | 1e-13 | -30.165454 | 24.39% | 0.62% | motif file (matrix) |
